# Supplementary material for: Subjective and Objective Cognitive Impairments in Non-Hospitalized Persons 9 Months after SARS-CoV-2 Infection
Source: Viruses. 2023 Jan 16;15(1):256. doi: 10.3390/v15010256 (PMC9865483; doi:10.3390/v15010256)
Supplement: Supplementary file 1 [file viruses-15-00256-s001.zip › Supplementary Table S1.pdf]

**Table S1.** Multivariable linear regression models for the cognitive tests WAIS-IV, RWT, SCWT-W, SCWT-C and SCWT-CW.

| | WAIS-IV | | | RWT | | | SCWT - W | | | SCWT – C | | | SCWT - CW | | |
|---|---|---|---|---|---|---|---|---|---|---|---|---|---|---|---|
| **Adjusted $R^2$** | **0.08** | | | **0.13** | | | **0.14** | | | **0.02** | | | **0.04** | | |
| **Variable** | **β** | **95% CI** | **p-value** | **β** | **95% CI** | **p-value** | **β** | **95% CI** | **p-value** | **β** | **95% CI** | **p-value** | **β** | **95% CI** | **p-value** |
| Age | -0.01 | -0.02 – 0.01 | 0.7738 | 0.71 | 0.48 – 0.94 | <.0.0001 | 0.16 | 0.12 – 0.21 | <.0.0001 | 0.04 | -0.03 – 0.11 | 0.2279 | 0.05 | 0.01 – 0.11 | 0.0398 |
| Female sex | -0.62 | -1.10 - -0.13 | 0.0125 | 3.02 | -3.72 – 9.75 | 0.3787 | 0.18 | -1.27 – 1.62 | 0.8105 | 1.34 | -0.59 – 3.27 | 0.1726 | 1.97 | 0.46 – 3.49 | 0.0108 |
| School education ≤ 9 years | -1.38 | -2.02 - -0.74 | <.0001 | -17.17 | -26.13 - -8.20 | 0.0002 | -3.10 | -5.02 - 1.18 | 0.0016 | -2.81 | -5.37 - -0.26 | 0.0311 | -2.14 | -4.14 - -0.13 | 0.0370 |
| Follow-up time | 0.04 | -0.04 – 0.11 | 0.3316 | 0.93 | -0.10 – 1.96 | 0.0777 | -0.06 | -0.29 - 0.16 | 0.5698 | -0.19 | -0.49 – 0.10 | 0.1999 | -0.05 | -0.29 – 0.18 | 0.6417 |
| Sum of complaints | 0.07 | -0.02 – 0.17 | 0.1256 | 0.07 | -1.22 – 1.36 | 0.9196 | -0.23 | -0.51 – 0.05 | 0.1030 | -0.12 | -0.49 – 0.25 | 0.5184 | -0.28 | -0.57 – 0.01 | 0.0560 |
| Concentr. Problems | -0.47 | -1.29 – 0.36 | 0.2706 | -6.81 | -18.33 – 4.71 | 0.2458 | -0.15 | -2.61 – 2.31 | 0.9046 | -1.76 | -5.03 – 1.51 | 0.2904 | 0.38 | -2.19 – 2.95 | 0.7710 |
| Memory problems | -0.38 | -0.06 – 0.14 | 0.3515 | 6.19 | -5.07 – 17.44 | 0.2803 | 0.98 | -1.42 – 3.38 | 0.4227 | 0.79 | -2.40 – 3.98 | 0.6267 | -0.39 | -2.90 – 2.11 | 0.7582 |
| Depression (PHQ-9) | 0.04 | -0.06 – 0.14 | 0.4846 | 1.51 | 0.12 – 2.90 | 0.0332 | 0.23 | -0.07 – 0.53 | 0.1370 | 0.48 | 0.08 – 0.89 | 0.0191 | 0.32 | 0.01 – 0.64 | 0.0442 |
| Mental HRQOL (VR-12) | 0.03 | -0.00 – 0.06 | 0.1391 | 0.57 | 0.07 – 1.07 | 0.0266 | 0.14 | 0.03 – 0.25 | 0.0113 | 0.13 | -0.01 – 0.28 | 0.0702 | 0.08 | -0.04 – 0.19 | 0.1764 |
| PTSD (IES-R) | -0.18 | -0.42 – 0.07 | 0.1509 | -3.76 | -7.14 - -0.38 | 0.0292 | -0.57 | -1.29 – 0.16 | 0.1249 | -0.69 | -1.65 – 0.28 | 0.1635 | -0.62 | -1.38 – 0.13 | 0.1061 |
| Disturbance of smell | -0.04 | -0.78 – 0.69 | 0.9096 | -2.65 | -12.91 – 7.61 | 0.6122 | 0.71 | -1.48 – 2.90 | 0.5237 | 1.32 | -1.59 – 4.23 | 0.3731 | 2.35 | 0.06 – 4.63 | 0.0441 |

| | | | | | | | | | | | | | | | |
|---|---|---|---|---|---|---|---|---|---|---|---|---|---|---|---|
| Disturbance of taste | 0.24 | -0.55 – 1.03 | 0.5496 | 2.12 | -8.90 – 13.14 | 0.7052 | 0.12 | -2.23 – 2.48 | 0.9174 | -1.50 | -4.63 - 1.63 | 0.3457 | -1.19 | -3.65 – 1.27 | 0.3411 |
| Headache | 0.16 | -0.47 – 0.80 | 0.6132 . | 0.07 | -8.72 – 8.86 | 0.9873 | 1.01 | -0.88 – 2.90 | 0.2926 | 1.56 | -0.95 – 4.07 | 0.2217 | 1.14 | -0.83 – 3.11 | 0.2551 |
| Vertigo | 0.47 | -0.42 – 1.37 | 0.3004 | -2.09 | -14.56 – 10.39 | 0.7424 | 0.57 | -2.11 – 3.24 | 0.6780 | -0.83 | -4.39 – 2.73 | 0.6478 | -1.92 | -4.72 – 0.87 | 0.1772 |
| Sleep problems | -0.04 | -0.70 – 0.61 | 0.8999 | -4.35 | -13.45 – 4.74 | 0.3469 | 0.96 | -0.99 – 2.92 | 0.33 | 0.01 | -2.59 – 2.61 | 0.9959 | 1.15 | -0.89 – 3.20 | 0.2669 |

WAIS-IV: Wechsler Adult Intelligence Scale; RWT: Regensburger Wortflüssigkeitstest (verbal fluency); SCWT: Stroop Color and Word Test - word (W), color (C), color – words (CW); CI: Confidence interval; PHQ-9: Patient Health Questionnaire; HRQOL: Health-related quality of life; VR-12: Veterans RAND 12-Item Health Survey, Mental Summary Scale; PTSD: Post-traumatic Stress Disorder; IES-R: Impact of Event Scale revised.
