# Supplementary material for: Subjective and Objective Cognitive Impairments in Non-Hospitalized Persons 9 Months after SARS-CoV-2 Infection
Source: Viruses. 2023 Jan 16;15(1):256. doi: 10.3390/v15010256 (PMC9865483; doi:10.3390/v15010256)
Supplement: Supplementary file 1 [file viruses-15-00256-s001.zip › Supplementary Table S2.pdf]

Table S2. Multivariable logistic regression models for the dependent variables concentration problems and memory problems at follow-up.

		Concentration problems			Memory problems		
**Variable**	**Ref.**	**OR**	**95% CI**	**p-value**	**OR**	**95% CI**	**p-value**
Age		0.97	0.94 – 0.99	0.0142	0.99	0.96 – 1.01	0.2803
Female sex	male	1.00	0.45 – 2.24	0.9946	0.94	0.44 – 2.01	0.8782
School education ≤ 9 years	> 9 years	2.20	0.80 – 6..05	0.1287	1.53	0.58 – 4.03	0.3857
Follow-up time		1.00	0.88 – 1.13	0.9431	1.06	0.94 – 1.18	0.3399
Sum of complaints		1.59	1.38 – 1.85	<.0001	1.32	1.17 – 1.48	<.0001
Concentration problems[1] (yes)	no	11.81	4.75 – 29.32	<.0001	-	-	-
Memory problems[1] (yes)	no	-	-	-	7.09	3.22 – 15.61	<.0001
Depression (PHQ-9)		1.15	1.00 – 1.33	0.0452	1.25	1.09 – 1.44	0.0010
Mental HRQOL (VR-12)		1.00	1.00 – 1.05	0.9706	1.03	0.98 – 1.08	0.2432
PTSD (IES-R)		0.93	0.65 – 1.33	0.7063	0.92	0.66 – 1.28	0.6164
Disturbance of smell (yes)	no	0.23	0.07 – 0.78	0.0186	0.33	0.10 – 1.02	0.0546
Disturbance of taste (yes)	no	1.46	0.45 – 4.76	0.5338	2.11	0.69 – 6.42	0.1887
Headache (yes)	no	0.94	0.39 – 2.29	0.8932	0.56	0.24 – 1.34	0.1949
Vertigo (yes)	no	0.62	0.18 – 2.16	0.4562	1.08	0.34 – 3.49	0.8935
Sleep problems (yes)	no	0.49	0.18 – 1.33	0.1620	0.72	0.29 – 1.76	0.4684

OR: Odds Ration; CI: Confidence interval; PHQ-9: Patient Health Questionnaire; HRQOL: Health-related quality of life; VR-12: Veterans RAND 12-Item Health Survey, Mental Summary Scale; PTSD: Post-traumatic Stress Disorder; IES-R: Impact of Event Scale revised; [1] acute COVID-19 phase.
