# Supplementary material for: Subjective and Objective Cognitive Impairments in Non-Hospitalized Persons 9 Months after SARS-CoV-2 Infection
Source: Viruses. 2023 Jan 16;15(1):256. doi: 10.3390/v15010256 (PMC9865483; doi:10.3390/v15010256)
Supplement: Supplementary file 1 [file viruses-15-00256-s001.zip › Supplementary Table S3.pdf]

Table S3: Multivariable linear regression models for the cognitive tests (with acute symptoms as covariables)

	WAIS-IV			RWT			SCWT - W			SCWT – C			SCWT - CW		
**Adjusted R^2**	**0.09**			**0.13**			**0.14**			**0.02**			**0.04**		
**Variable**	**β**	**95% CI**	**p-value**	**β**	**95% CI**	**p- value**	**β**	**95% CI**	**p-value**	**β**	**95% CI**	**p- value**	**β**	**95% CI**	**p-value**
Age	-0.00	-0.02 – 0.02	0.9632	0.71	0.48 – 0.94	<.0.0001	0.17	0.12 – 0.22	<.0.0001	0.05	-0.02 – 0.11	0.1782	0.06	0.01 – 0.11	0.0273
Female sex	-0.65	-1.13 - -0.17	0.0079	2.50	-4.19 – 9.19	0.4629	0.27	-1.18 – 1.70	0.7168	1.42	-0.50 – 3.33	0.1456	1.94	0.43 – 3.45	0.0120
School education ≤ 9 years	-1.41	-2.06 - -0.77	<.0001	-16.82	-25.75 - -7.89	0.0002	-3.22	-5.14 - 1.30	0.0011	-2.83	-5.38 - -0.28	0.0296	-1.97	-3.98 - -0.04	0.0549
Follow-up time	0.04	-0.04 – 0.11	0.3305	0.91	-0.11 – 1.94	0.0814	-0.08	-0.30 -0.14	0.4692	-0.23	-0.53 – 0.06	0.1162	-0.09	-0.32 – 0.15	0.4681
Sum of complaints	0.10	-0.03 – 0.17	0.0076	-0.14	-1.16 – 0.87	0.7800	-0.11	-0.33 -0.11	0.3267	-0.06	-0.35 – 0.26	0.7099	-0.18	-0.41 – 0.05	0.1333
Concentr. Problems	-0.50	-1.31 – 0.32	0.2297	-5.25	-16.57 – 6.06	0.3616	-0.43	-2.85 – 2.00	0.7251	-1.68	-4.88 – 1.53	0.3045	0.08	-2.46 – 2.61	0.9519
Memory problems	-0.37	-1.17 – 0.43	0.3612	5.83	-5.27 – 16.93	0.3020	0.95	-1.42 – 3.33	0.4301	0.45	-2.70 – 3.60	0.7786	-0.68	-3.17 – 1.81	0.5901
Depression (PHQ-9)	0.03	-0.07 – 0.13	0.5380	1.39	0.02 – 2.77	0.0476	0.25	-0.04 – 0.25	0.0071	0.48	0.08 – 0.88	0.0197	0.32	0.01 – 0.64	0.0455
Mental HRQOL (VR-12)	0.03	-0.01 – 0.06	0.1244	0.52	0.03 – 1.02	0.0398	0.15	0.03 – 0.25	0.0113	0.13	-0.01 – 0.28	0.0716	0.09	-0.03 – 0.20	0.1363
PTSD (IES-R)	-0.17	-0.41 – 0.08	0.1825	-3.76	-7.19 - -0.33	0.0317	-0.51	-1.25 – 0.23	0.1739	-0.51	-1.49 – 0.46	0.3016	-0.54	-1.31 – 0.23	0.1710

Disturbance of smell[1]	-0.10	-0.84 – 0.64	0.7878	1.86	-8.46 – 12.19	0.7231	0.95	-1.28 – 3.19	0.4023	1.75	-1.21 – 4.71	0.2449	2.48	0.14 – 4.81	0.0380
Disturbance of taste[1]	0.17	-0.56 – 0.91	0.6397	-3.74	-13.92 – 6.45	0.4710	-0.03	-2.24 – 2.17	0.9754	-0.98	-3.90 -1.94	0.5106	-1.98	-4.29 – 0.33	0.0928
Headache[1]	0.49	-0.03 – 1.01	0.0645.	3.83	-3.44 – 11.09	0.9873	-0.44	-1.99 – 1.12	0.5793	-0.05	-2.11 – 2.02	0.9639	0.39	-1.24 – 2.02	0.6381
Vertigo[1]	-0.18	-0.70 – 0.34	0.4908	-4.38	-11.59 – 2.83	0.2330	-0.30	-1.85 – 1.25	0.7046	-0.97	-3.04 – 1.09	0.3541	-0.47	-2.10 – 1.16	0.5712
Sleep problems[1]	-0.29	-0.84 – 0.27	0.3075	-1.76	-9.44 – 5.93	0.6530	0.07	-1.60 – 1.73	0.9375	-1.45	-3.66 – 0.76	0.1974	-0.64	-2.38 – 1.11	0.4746

WAIS-IV: Wechsler Adult Intelligence Scale; RWT: Regensburger Wortflüssigkeitstest (verbal fluency); SCWT: Stroop Color and Word Test - word (W), color (C), color – words (CW); CI: Confidence interval; PHQ-9: Patient Health Questionnaire; HRQOL: Health-related quality of life; VR-12: Veterans RAND 12-Item Health Survey, Mental Summary Scale; PTSD: Post-traumatic Stress Disorder; IES-R: Impact of Event Scale revised

[1]During the acute COVID-19 phase
