# Supplementary material for: Subjective and Objective Cognitive Impairments in Non-Hospitalized Persons 9 Months after SARS-CoV-2 Infection
Source: Viruses. 2023 Jan 16;15(1):256. doi: 10.3390/v15010256 (PMC9865483; doi:10.3390/v15010256)
Supplement: Supplementary file 1 [file viruses-15-00256-s001.zip › Supplementary Table S4.pdf]

Table S4: Multivariable logistic regression models for the dependent variables concentration problems and memory problems (with acute symptoms as covariables)

		Concentration problems			Memory problems		
**Variable**	**Ref.**	**OR**	**95% CI**	**p-value**	**OR**	**95% CI**	**p-value**
Age		0.96	0.94 – 0.99	0.0077	0.99	0.96 – 1.01	0.2803
Female sex	male	0.90	0.41 – 1.98	0.7953	0.83	0.40 – 1.72	0.6070
School education ≤ 9 years	> 9 years	1.88	0.68 – 5.19	0.2234	1.43	0.55 – 3.71	0.4639
Follow-up time		1.00	0.89 – 1.13	0.9666	1.07	0.96 – 1.20	0.2397
Sum of complaints		1.50	1.32 – 1.70	<.0001	1.27	1.16 – 1.40	<.0001
Concentration problems[1] (yes)	no	14.94	5.62 – 39.74	<.0001	-	-	-
Memory problems[1] (yes)	no	-	-	-	7.64	3.32 – 17.58	<.0001
Depression (PHQ-9)		1.13	0.98 – 1.29	0.0911	1.25	1.09 – 1.42	0.0012
Mental HRQOL (VR-12)		1.00	0.94 – 1.04	0.6341	1.02	0.97 – 1.07	0.4470
PTSD (IES-R)		0.99	0.70 – 1.41	0.9736	0.93	0.67 – 1.29	0.6635
Disturbance of smell[1] (yes)	no	0.95	0.28 – 3.30	0.9382	0.77	0.25 – 2.36	0.6511
Disturbance of taste[1] (yes)	no	1.97	0.56 – 6.92	0.2880	1.24	0.41 – 3.69	0.7060
Headache[1] (yes)	no	0.50	0.19 – 1.31	0.1599	0.96	0.42 – 2.21	0.9240
Vertigo[1] (yes)	no	0.80	0.36 – 1.77	0.5831	0.78	0.37 – 1.67	0.5259
Sleep problems[1] (yes)	no	0.70	0.30 – 1.61	0.4010	0.77	0.36 – 1.67	0.5122

OR: Odds Ration; CI: Confidence interval

PHQ-9: Patient Health Questionnaire; HRQOL: Health-related quality of life; VR-12: Veterans RAND 12-Item Health Survey, Mental Summary Scale; PTSD: Post-traumatic Stress Disorder; IES-R: Impact of Event Scale revised

[1]acute COVID-19 phase
